# Mitigating Extreme Summer Heat Waves with the Optimal Water-Cooling Island Effect Based on Remote Sensing Data from Shanghai, China

**DOI:** 10.3390/ijerph19159149

**Published:** 2022-07-27

**Authors:** Hongyu Du, Fengqi Zhou

**Affiliations:** Institute of Ecology and Sustainable Development, Shanghai Academy of Social Sciences, No. 7, Lane 622, Huaihaizhong Road, Huangpu District, Shanghai 200020, China; zhoufengqi10@126.com

**Keywords:** water cooling island effect (WCI), landscape pattern, remote sensing (RS), regress analysis

## Abstract

Due to the progress in global warming, the frequency, duration and intensity of climate extremes are increasing. As one of these extremes, heat waves influence the well-being of human beings and increase societies’ energy consumption. The Water-Cooling Island (WCI) effect of urban water bodies (UWBs) is important in urban heat wave mitigation. In this paper, the impact of WCI, especially the landscape pattern of the surrounding area, was explored. The results indicate that water bodies with a larger total area and simpler shape have a longer cooling effect. In the areas surrounding UWBs, a lower percentage or discrete distribution of impervious surfaces or green land provide a longer cooling effect. The amplitude of WCI is mainly decided by the impervious surface in the surrounding areas. A lower percentage or discrete distribution of impervious surfaces or green land leads to a smaller-amplitude WCI. The gradient is impacted by the shape of the UWB and surrounding green land. A complex shape and discrete distribution of green land lead to a higher gradient of WCI. The linear regress model was significant in terms of WCI range and gradient, while the model of WCI amplitude was not significant. This indicates that WCI is directly decided by impact factors through gradient and range. The conclusions provide a methodology for WCI prediction and optimization, which is important when mitigating summer heat waves.

## 1. Introduction

Due to the progress of global warming, climate extremes are presented more frequently [1,2,3,4]. One such extreme is heat waves, which have serious negative effects on urban life [5,6]. This is especially true in constructed areas, where the Urban Heat Island (UHI) effect enhances the problem [7]. It has been proved by clinical trials that temperatures above 28 °C lead to anxiety, depression, hypomnesis and dyspepsia. If the temperature rises above 34 °C, the incidences of the respiratory system, cardiovascular and cerebrovascular diseases and corresponding mortality dramatically increase [8]. In the summer of 2003, around 35,000 deaths were reported as a result of the heat wave in Europe [9]. In 2022, the highest historical temperature was reported in most European countries. Portugal reached 47 °C, France reported 42 °C and temperatures of 40.3 °C were reported in the UK. A food crisis is estimated to follow this extreme climate. Around 900 million people were affected by the heat wave in China, with many deaths reported due to thermal radiation sickness. The IPCC report indicated that the intensity, frequency and duration of heat waves would significantly increase [10]. Thus, the persistent mitigation of extremely high temperatures in summer is a research priority.

The fundamental methodology for heat wave mitigation is to continuously reduce the emission of greenhouse gases [11]. Then, global warming, which causes the increment in climate extremes, can be slowed down or eliminated. However, this topic needs global cooperation, political debates and wide evolution in all industries [12,13]. The more direct way is to make full use of the Water-Cooling Island (WCI) effect, especially in riparian and coastal cities [14,15]. In constructed areas, urban water bodies (UWB) have higher thermal inertia compared to artificial impervious surfaces [16]. In addition, the evaporation effect under solar radiation dissipates the absorbed heat [17]. Therefore, UWBs have a lower temperature in summer than their surrounding areas [18]. This phenomenon is defined as the WCI effect [19]. To mitigate heat waves, it is infeasible to simply enlarge the water bodies, as the land resources are limited. Therefore, the WCI impact factors need to be found, and the interaction mechanism needs to be revealed. Then, the water bodies can be optimally designed and planned in the city to maximize the WCI within limited spaces.

Previously in this research field, the most-discussed impact factor was the UWB size, as the amount of water directly influences the total thermal inertia of UWBs. For example, using a step-wise regress analysis based on remote-sensing data, Yang, Meng [20] revealed that the average size of the water body is the most significant impact factor in WCIs. Plenty of studies reached similar conclusions.

The second most discussed topic is the shape of the UWB. For example, Sun, Chen [21] carried out an investigation of WCI intensity based on the ASTER images of UWB in Beijing. The results showed that WCI is also significantly correlated with the Landscape Shape Index (LSI). By using a statistical analysis of UWBs in Shanghai, Yue and Xu [22] reached similar results; a complex UWB outline leads to a strong WCI.

According to the principle of thermodynamics, other potential impact factors include season, water dynamics and combined green land and wind conditions. These all influence WCI by changing the heat exchange features. For example, Yang, Meng [20] indicated that dynamic water bodies lead to a stronger WCI than static ones. This is due to the continuous heat dissipation caused by dynamic water. However, the water temperature of an upper stream may influence the validity of the result. This aspect was not discussed in the research. Hathway and Sharples [23] carried out research on WCI using the field survey method over spring and summer for a river in Sheffield, UK. The results show that WCI is stronger in spring than in summer.

In addition to the size, shape, season and dynamic features, the space feature around the UWB also has a direct influence on WCI. For example, Sun, Chen [21] observed that the WCI in Beijing is related to the distance between the water body and the city center. Meanwhile, Hathway and Sharples [23] revealed that WCI is related to the city’s arrangement on the river banks. However, direct impact factors are not revealed in either piece of research. Wu and Zhang [24] further researched the WCI effect beside Suzhou Bay; it was concluded that the land use beside the water body influences the LST distribution.

All the above-mentioned research indicates that the WCI of UWB is a complex model with multiple impact factors. More case studies are necessary to validate the current conclusions. The impact factors from areas around UWB have not yet been discussed in detail. Furthermore, most research in this field concentrates only on the correlation between impact factors and WCI; WCI has yet to be quantitively modeled.

By using a synthetic application of Remote Sensing (RS), Atmosphere Transfer Modeling (ATM) and Pearson correlation analysis, based on case data from Shanghai, China, this research aimed to reveal the relationship between impact factors and WCI in summer. Indicators of the surrounding areas of UWB were included. Then, quantitative regress models of WCI were built and validated. These conclusions and models can serve as good predictors of the benefits that WCIs receive from the specific design and planning of UWB. Then, the influence of a summer heat wave can be mitigated, which benefits the health and well-being of citizens.

## 2. Research Region

**Figure 1:**
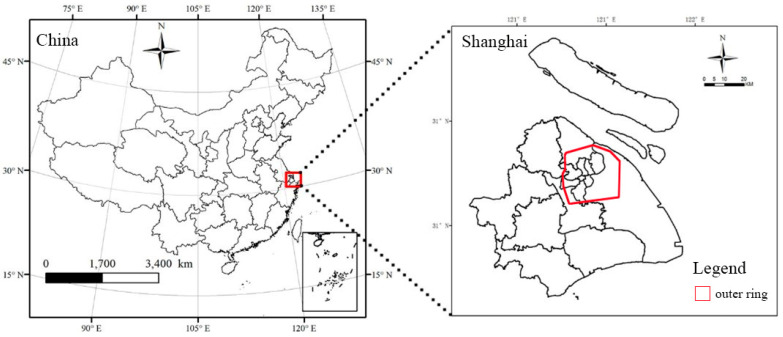
Location of Shanghai, China.

Shanghai, with a subtropical monsoon climate and a total area of 6340.5 km^2^, is located on the east coast of China (Figure 1). In recent years, extreme summer temperatures have continuously increased in both intensity and frequency [25]. In 2013, from June to August, the number of high-temperature days (peak temperature ≥ 35 °C) reached 47. On 21 July 2017, the air temperature at Xujiahui National Meteorological Station reached 40.9 °C, the highest recorded temperature in history. On 13 July, the same record was reached again. These extreme temperatures threatened the health of citizens and caused an increase in the consumption of artificial energy. Mitigating summer heat waves is a common concern among citizens and the government in Shanghai. Furthermore, as one of the best-developed cities in China, the mega-city, with a population of 24 million, has sophisticated land-management techniques, especially in the central areas. The serious UHI effect enhances the consequences of heat waves [26]. Meanwhile, as it sits at the Yangtze River delta, the city has rich water resources. Mitigating heat waves using UWB is an emerging public requirement.

## 3. Methodology

### 3.1. Selection and Quantification of Impact Factors

The selected impact factors and corresponding mathematic indicators in this research are listed in Table 1 [25]. Since the impact factors from the surrounding area of the UWB are stressed in this research, four new factors are introduced in Table 1. They are the percentage of impervious surfaces (PI), percentage of green land (PG), Mean Nearest Neighbor index of green land (MNNg) and Mean Nearest Neighbor index of impervious (MNNi). They describe the land-use condition and landscape pattern of the areas, respectively. PI and PG indicate the ratio of two typical land use, which has a significant thermal dynamic contribution to the environment. A high PI or PG means that more impervious or green land is distributed in the surrounding area of UWB. MNNg and MNNi indicate the type of distribution. A high MNNg means that the green land patches around UWB are evenly randomly distributed, while a low MNNg means that the patches are gathered.

In order to carry out the study, the impact factors need to be quantified. Therefore, high-resolution Google Earth images were introduced into Fragstats 4.2 software (University of Massachusetts Amherst, Amherst, MA, USA) to abstract the features of water bodies and the landscape pattern of their surrounding area. In this method, all the remaining impact indicators are calculated.

The LSI indicates the complexity of the landscape patch’s shape. A larger LSI means that the shape is more complex. This can be calculated using Equation (1) [27].

(1) $$LSI=\frac{P}{2\sqrt{\pi \times A}}$$

In the equation, P is the perimeter of the landscape patch, and A is the area of the patch. LSI=1 means the shape is circular, while LSI=1.13 indicates a square shape.

MNNg and MNNi are the landscape pattern indicators representing the connectivity and distribution of landscape patches [28]. A larger number means a more discrete patch distribution. They can be calculated using Equation (2).

(2) $$MNN=\frac{{\bar{D}}_{O}}{{\bar{D}}_{E}}$$

${\bar{D}}_{O}$ is the mean average distance between the focus patch and the other patches and can be calculated using Equation (3).

(3) $${\bar{D}}_{O}=\frac{\sum_{i=1}^{n}d_{i}}{n}$$

${\bar{D}}_{E}$ is the mean average distance between elements if they are uniformly distributed and can be calculated using Equation (4).

(4) $${\bar{D}}_{E}=\frac{0.5}{\sqrt{\frac{n}{A}}}$$

$d_{i}$ indicates the distance between elements with a number index i and their closest element. N indicates the total number of elements in the area. A means the total area of the concerned space. A higher MNN means that the patches are more randomly distributed in the defined area. A lower MNN, especially one lower than 1, means that the patches are agminated.

Season, water dynamics and wind were not considered due to the following reasons:

(1) WCI in summer daytime is used, as the focus of this study is mitigating summer heat waves using WCI.
(2) Although the wind has a valid influence on WCI, it is difficult to control this factor in research influenced by complex real-world uncertainties.
(3) Linear UWB usually leads to different WCI results in different sections because the space is too wide. This makes the abstraction of impact factors difficult.

As the urban water bodies in Shanghai are normally less than 2 m deep, the depth of UWB is also not considered.

### 3.2. Acquisition of Temperature Data

Two methods were mainly used to acquire the temperature data: field meteorological measurement and RS [26,29]. Field meteorological measurements focus on the air temperature. This is the only choice if the focus is urban heat-island air effects. The main disadvantage is the limited number of testing points; polygon regression is required to obtain a complete spatial temperature distribution [23,30]. RS has the advantage of data synchronization and wide distribution [31]. The accuracy increases with continuous technical improvements.

In this research, the land-cover conditions around the UWB were quite complicated. There were green lands, dense buildings and streets with traffic. It was quite difficult to apply meteorological field measurements. As the wind was eliminated, the surface WCI was acceptable. Then, the RS method was finally applied.

Referring to the three preconditions defined in Section 3.1, the Landsat RS data for Shanghai on the dates shown in Table 2 were selected as they satisfy the following requirements:

(1) The weather should be clear with few clouds. This provides a clear RS image, and missing pixels can be avoided;
(2) The season should be summer, when WCI is the most important, as only summer WCI is discussed in this study;
(3) There should be no wind or a gentle breeze.

Then, based on the RS figure, the Land Surface Temperature (LST) can be acquired using Atmosphere Transfer Modeling (ATM) [32]. This method was introduced in multiple previous studies in this field [33]. The contents are introduced below for convenience.

First, ENVI5.1 was used to preprocess the raw images, including radiometric calibration, atmospheric correction, cutting and splicing. For atmospheric correction, the atmospheric radiation process was simulated by MODTRAN 4.0 software (Berk, A., et al., Burlington, MA, USA). The simulation model is based on the radiative transfer function (Equation (5)). By using simulation, atmospheric downward radiance $L_{atm,i}↓$, upward radiance $L_{atm,i}↑$ and transmissivity τ can be estimated. Then, with the given land surface emissivity ε, the $B\left(T_{s}\right)$ in Equation (5) can be calculated. LST (*T_s_*) can be calculated using Equation (6).

(5) $$L_{sensor,i}=\tau_{i}\varepsilon_{i}B\left(T_{s}\right)+\left(1-\varepsilon_{i}\right)\tau_{i}L_{atm,i}↓+L_{atm,i}↑$$

(6) $$B\left(T_{s}\right)=\frac{c_{1}}{\lambda^{5}\left(e^{\left(\frac{c_{2}}{\lambda T_{s}}\right)}-1\right)}$$

$L_{sensor,i}$ in Equation (5) is the radiation intensity (Wm^−2^sr^−1^μm^−1^) of wave band i, measured by a satellite sensor. This can be obtained by the gray value of raw images, according to Equation (7).

(7) $$L_{sensor,i}=gain\times QCAL+offset$$

QCAL is the gray value, G is the gain value in wave band i and *offset* is the deviation value in wave band i. For the two thermal infrared bands of Landsat-8, both gains are identical (0.0003342), and so are the offsets (0.1).

In Equation (6), $B\left(T_{s}\right)$ is the black body radiation intensity gained by Plank radiation function; $c_{1}$ and $c_{2}$ are radiation constants. The values are 1.19104356 × 10^8^ Wm^−2^sr^−1^μm^4^ and 1.4387685 × 10^4^ μmK, respectively. *λ* is wavelength (μm). Equation (6) can be transformed into Equation (8).

(8) $$T_{s}=\frac{K_{2}}{\ln\left(1+\left(\frac{K_{1}}{B\left(T_{s}\right)}\right)\right)}$$

$K_{1}$ (Wm^−2^sr^−1^μm^−1^) and $K_{2}$ are the preset constants before launch. The values of TIRS data for Landsat-8 are listed in Table 3.

### 3.3. Quantification of WCI

**Figure 2:**
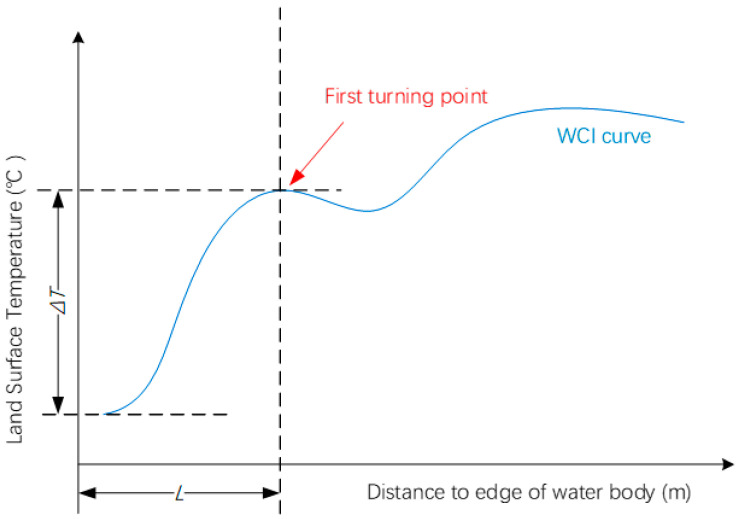
WCI quantification curve: vertical axel indicates land surface temperature; longitudinal axel indicates the distance from the edge of the water body.

The definition of WCI is a lower temperature in UWBs and their surrounding areas compared to the urban background environment [34]. Therefore, the direct quantification of WCI is the amplitude of this temperature difference (ΔT). Another consideration is the size of the WCI-covered area. We used the range of WCI (L) as a quantification indicator. The last one was the temperature gradient (G). According to previous research by other authors, the three WCI indicators can be acquired by setting up a two-dimensional temperature curve (Figure 2) [21]. The vertical axel indicates the temperature, while the horizon axel indicates the distance from the UWB. The first turning point or asymptote (no turning point) of this temperature curve indicates the background temperature. ΔT can be calculated as the difference on the vertical axel. L can be calculated as the difference on the horizon axel. G can be calculated as the ratio between ΔT and L.

### 3.4. Methodology of Statistical Analysis

The statistical methods applied in this research were Pearson correlation analysis and Least Square Linear Regress (LSLR) analysis. The Pearson correlation method was used to reveal the relationship between WCI and impact factors, especially the correlation between WCI (ΔT, L and G) and the indicators of surrounding areas (PI, PG, MNNg and MNNi). LSLR was used to set up a regression model of WCI. Some samples were not included in the LSLR analysis because the left samples were used to validate the built models.

### 3.5. Selection of Research Samples

**Figure 3:**
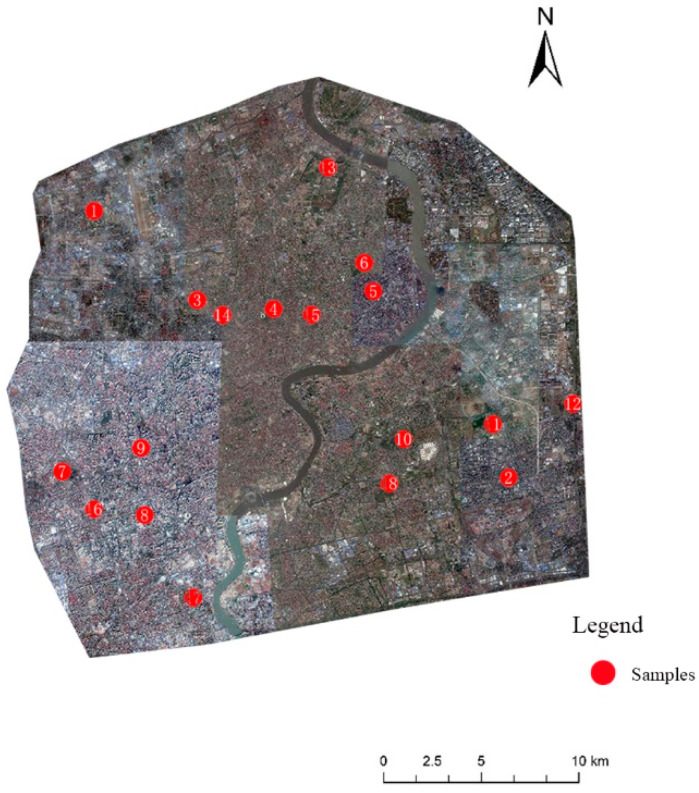
Location of the 18 water bodies in Shanghai (marked in red numbers).

In this study, 18 planner UWBs with an area greater than 1 ha in the outer ring road of Shanghai were selected. The areas within the outer ring road were all constructed regions, which means that the UHI effect is significant. The UWB locations are shown in Figure 3.

## 4. Result

### 4.1. Overview of Data

**Figure 4:**
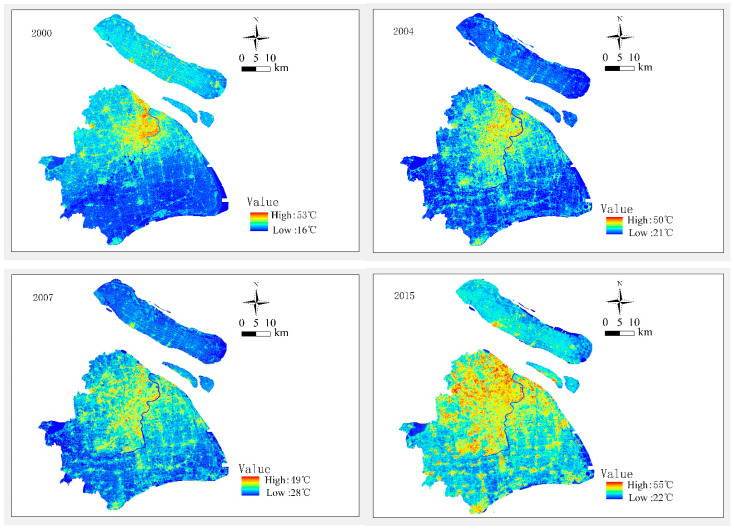
The LST retrieval results for Shanghai-based on Landsat data in the years 2000, 2004, 2007 and 2015.

The results of LST retrieval using the ATM method are shown in Figure 4. The abstracted WCI indicators, together with the average LST values, are shown in Table 4. The calculated impact indicator values are shown in Table 5.

### 4.2. Correlation between Impact Factors

First, the relationship between the impact factors was analyzed to eliminate the strong correlation. The result is shown in Table 6.

Table 6 indicates that PI and PG are significantly negatively correlated. This is due to the surrounding area of the water body, which is mainly formed of vegetation and impervious surfaces. Except for this, the correlation between other factors is not quite significant. Thus, PG is eliminated in the following analysis.

### 4.3. Analysis of WCI Impact Factors

The correlation between WCI indicators and impact factors is shown in Table 7.

According to the results, L is significantly positively correlated with S, MNNi and MNNg, and significantly negatively correlated with LSI and PI. That means that UWB with a larger total area or simpler shape has a longer cooling effect. In the surrounding areas, a lower percentage of impervious surfaces leads to a longer cooling effect. Meanwhile, more discrete distributed impervious surfaces or more green land can lengthen the cooling effect.

The results also show that ΔT is significantly negatively correlated with PI and significantly positively correlated with MNNi. This indicates that the amplitude of WCI is mainly decided by the impervious surface in the surrounding areas. A lower percentage or discrete distribution of impervious surfaces decreases the WCI amplitude. ΔT is slightly positively correlated with MNNg. This means that a more discrete distribution of green land also leads to an increased WCI amplitude.

G is positively correlated with LSI and negatively correlated with MNNg. The positive correlation indicates that a complicated UWB outline leads to a higher gradient of WCI. The discrete distribution of green land in the surrounding area of UWB leads to a smaller gradient.

### 4.4. Regress Model of WCI

In this section, a regression model of WCI was built. Multiple-variation LSLR analysis was applied. In order to evaluate the significance of the regression models, except for the significance check indicators, the data in 2015 for Samples 13–18 were taken from LSLR analysis in the result-checking group.

#### 4.4.1. Range of WCI

According to Section 4.3, L is significantly correlated with S, LSI, PI MNNg and MNNi. A linear regression model of L is shown in Equation (9). *A*_0_ is constant. *A*_1_–*A*_5_ are the weight factors of each variation.

L = *A*_0_ + *A*_1_ × S + *A*_2_ × LSI + *A*_3_ × PI + *A*_4_ × MNNg + *A*_5_ × MNNi (9)

Then, the regression calculation table in SPSS is shown in Table 8.

According to Table 8, the model is shown in Equation (10).

L = 2.050 + 0.014 × S − 0.134 × LSI − 1.282 × PI − 0.220 × MNNg + 0.109 × MNNi(10)

**Figure 5:**
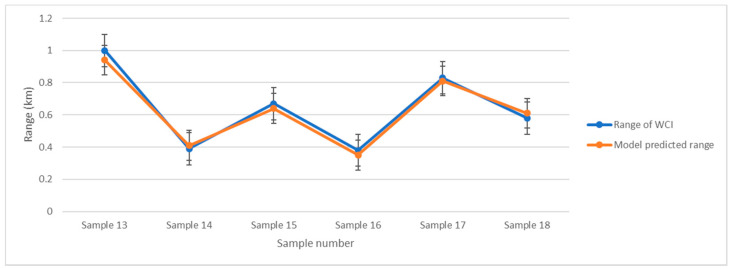
L Regression model validation of WCI range by estimating the other 6 samples.

The validation results for the remaining six samples are shown in Figure 5. The correlation factor between two results is 0.993 (*p* < 0.001). This means the model can indicate the L of WCIs.

#### 4.4.2. Amplitude of WCI

According to Section 4.3, ΔT is significantly correlated with PI and MNNi. A linear regression model of ΔT is shown in Equation (11). *A*_0_ is constant. *A*_1_ and *A*_2_ are the weight factors of each variation.

ΔT = *A*_0_ + *A*_1_ × PI + *A*_2_ × MNNg + *A*_3_ × MNNi (11)

The regression calculation table in SPSS is shown in Table 9.

According to Table 9, the model is shown in Equation (4).

ΔT = 4.691 − 3.663 × PI + 0.247 × MNNg + 0.856 × MNNi(12)

**Figure 6:**
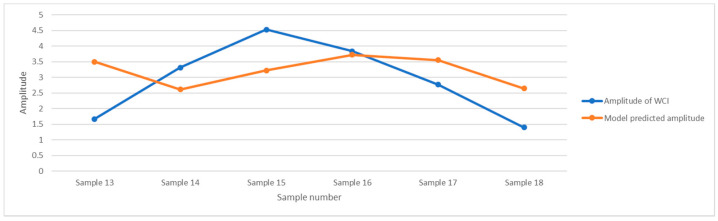
Regression model validation of WCI amplitude obtained by estimating the other 6 samples.

The validation results for the remaining six samples are shown in Figure 6. The correlation between the two results is 0.107 (*p* < 0.84). The regression model is not significant.

#### 4.4.3. WCI Gradient

According to Section 4.3, G is significantly correlated with LSI. The second most significant impact factor is MNNg. Therefore, the linear regress model of G is shown in Equation (12). *A*_0_ is constant. *A*_1_ and *A*_2_ are the weight factors of each variation.

G = *A*_0_ + *A*_1_ × LSI + *A*_2_ × MNNg (13)

The regression calculation table in SPSS is shown in Table 10.

According to Table 10, the model is given by Equation (13).

G = 0.936 + 1.842 × LSI − 0.027 × MNNg(14)

**Figure 7:**
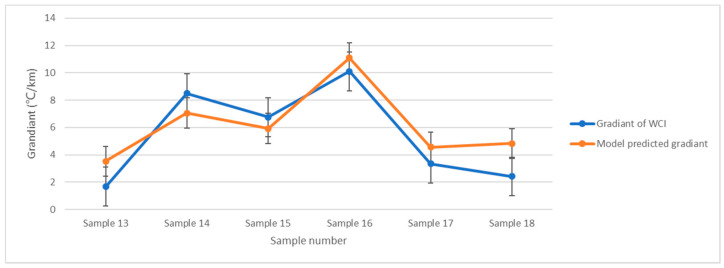
Regression model validation of WCI gradient by estimating the other 6 samples.

The validation results for the remaining six samples are shown in Figure 7. The correlation factor between two results is 0.910 (*p* < 0.012). This means the model can indicate the G of WCI.

## 5. Discussion

### 5.1. WCI and Self-Features

UWBs with a large total area or simple shape have a longer cooling effect. This coincides with the research results of Su, Huang [35] and Yang, Zhang [36]. UWBs with a similar depth but a larger total area can store more water and can cope with more heat. This leads to larger thermal inertia. Then, the transition buffer between the UWB and background thermal environment is longer, leading to a longer range of WCI. UWBs with a large LSI have increased contact with the surrounding environment. The evaporation and heat exchange are stronger, which also results in a longer range of WCI.

In the surrounding areas, a lower percentage of impervious surfaces leads to a longer cooling effect. This coincides with the conclusion of Shi, Deng [37]. The impervious surfaces around the UWB have a small thermal capacity. With the same solar radiation, the temperature increases. This increases the temperature of the UWB by heat exchange; meanwhile, the WCI in the area is also mitigated [38,39]. Then, the WCI range becomes smaller. For a similar reason, the WCI amplitude is mainly determined by the impervious surface in the surrounding areas. This coincides with the conclusion of Cao and Ding [40] and Sun, Chen [21].

The WCI gradient reflects two aspects of the thermal features. The first is the speed of the heat exchange between the UWB and the surrounding areas. The second is the thermal inertia of the surrounding area. A high level of inertia reflects that the surrounding environment is more easily affected by the WCI. A complicated UWB shape leads to a higher gradient of WCI. This is due to its increased contact with the surrounding areas, which speeds up heat exchange. In addition, a UWB with a large LSI can effectively slice the surrounding areas into smaller subareas with smaller thermal inertia. The WCI gradient is higher.

### 5.2. WCI and Landscape Pattern in the Surrounding Area

The result reflects that both the range and amplitude of WCI are positively correlated with MNNi. Thus, the landscape pattern indicators influence the WCI. A large MNNi leads to a dispersive distribution of the impervious landscape. Then, the high LST caused by the low thermal capacity of the impervious landscape is dissipated into small pieces. This effectively reduces the UHI in terms of both intensity and area. Thus, the L and ΔT are increased. This result can be compacted by the research results of Hathway and Sharples [23] and Sun, Chen [21]. The research of Sun, Chen [21] concluded that a UWB close to the city center has a smaller range and amplitude of WCI. The UWB close to the city center usually has a smaller MNNi. Therefore, to increase the intensity of WCI, it is recommended that UWBs are designed with dispersive distributed impervious surfaces.

On the other hand, the range and amplitude of WCIs are also positively correlated with MNNg; this indicates that the dispersive distribution of green land also enhances WCIs. With such a distribution, the hot zones of impervious surfaces cannot connect to each other, and the thermal environment is improved.

### 5.3. Regression Model of WCI

The linear regression models of L and G are significant. Their accuracy is also proved by validation. This shows that the relevant indicators are complete for the prediction of the WCI effect. However, the amplitude model is not as significant as L and G. The main factors that influence ΔT have yet to be fully explored. The result indicates that the impact factors first directly influence gradient and range. The amplitude is indirectly impacted.

### 5.4. Suggestion for WCI Design and Planning

According to the above analysis, from the perspective of making maximum use of WCIs to mitigate heat waves, it is recommended to use UWBs with a large LSI in the design and planning process and to connect urban spaces with irregular UWBs. The water body area should be as large as possible.

The percentage of impervious surfaces should be as low as possible. A good method is to use a vegetation buffer around the planned water body or on the riverbanks. In city planning, city spaces should be designed to have discrete distributed green land and impervious surfaces to take full advantage of the WCI effect.

### 5.5. Limitations and Future Work

Due to the research time and data availability, there are some limitations to this research.

First, although the statistical analysis is already significant for most of the topics, 18 water bodies are not enough to conduct an effective statistical analysis on the other topics.

Second, in this research, only planar water bodies were discussed. Linear water bodies were not included in the samples.

Additionally, only static WCI was analyzed, which means that wind, water dynamics, season, day and night were not included in the research scope. A simulation methodology should be applied in future studies.

Last, only a linear regression model was built. However, the ΔT model is not significant.

Future work should include more samples and introduce more impact factors to set up the ΔT regression model. Linear water bodies should be analyzed, and a more compact validation of the models should be completed.

## 6. Conclusions

In this paper, by using a statistical analysis of 18 UWBs in Shanghai, China, the WCI impact factors were revealed. The relationship between WCI and the surrounding area was particularly explored. A regression model of WCI range and gradient was set up and proved significant. The main conclusions are listed below.

Water bodies with a larger total area and simpler shape have a longer cooling effect. A lower percentage or discrete distribution of impervious surfaces or green land in surrounding areas also lengthens the cooling effect.

The amplitude of WCI is mainly determined by the impervious surface in the surrounding areas. A lower percentage or discrete distribution of impervious surfaces or green land lowers the WCI amplitude.

The gradient was impacted by the shape and amount of green land around the UWB. A complex shape and discrete distribution of green land increased the WCI gradient.

The regression model of the WCI range and gradient was significant. The model of WCI amplitude was not significant. This indicates that WCI is directly determined by impact factors such as gradient and range.

From the perspective of making maximum use of WCIs to mitigate summer heat waves, the use of irregular water bodies was recommended to connect urban spaces. The water bodies should be planned to be as large as possible, while the percentage of impervious surfaces should be as low as possible. It is recommended that green spaces are planned around water bodies to break impervious surfaces and strengthen the WCI.

The conclusions of this research can serve to optimize WCIs. The regression models can also provide support for WCI effect evaluations and predictions before construction.

## Figures and Tables

**Table 1:** WCI impact factors and indicators.

Impact Factors	Indicators
Size	Area (S)
Shape	landscape Shape Index (LSI)
Surrounding environment	Percentage of impervious (PI)
	Percentage of green land (PG)
	Mean nearest neighbor index of green land (MNNg)
	Mean nearest neighbor index of impervious (MNNi)

**Table 2:** Data: date and weather information.

Satellite/Sensor	Track No.	Date	Image Quality
Landsat-7 ETM+	118/38, 118/39	1 August 2000	No cloud
Landsat-5 TM	118/38, 118/39	19 July 2004	No cloud
Landsat-5 TM	118/38, 118/39	28 July 2007	No cloud
Landsat-8 TIRS	118/38, 118/39	3 August 2015	No cloud

**Table 3:** Preset constant values of TIRS dataset in Landsat-8 satellite.

Constant	TIRS1	TIRS2
*K*_1_	774.89	480.89
*K*_2_	1321.08	1201.4

**Table 4:** Quantification results of WCI indicators.

No.	Average LST (°C)				L (km)				ΔT (°C)				G (°C/km)			
	2000	2004	2007	2015	2000	2004	2007	2015	2000	2004	2007	2015	2000	2004	2007	2015
1	34.46	36.48	38.56	38.17	0.86	0.81	0.68	0.67	2.55	2.73	3.04	2.51	2.96	3.35	4.49	3.75
2	33.05	35.08	37.42	36.95	0.17	0.49	0.59	0.37	4.88	4.11	4.25	4.69	28.15	8.34	7.17	12.69
3	33.50	35.72	37.29	36.97	0.88	1.03	1.07	1.05	4.75	4.96	5.62	5.29	5.41	4.80	5.24	5.03
4	33.55	35.53	36.80	36.84	0.99	0.91	0.85	0.80	4.82	5.22	5.42	5.28	4.87	5.72	6.34	6.59
5	36.21	38.14	39.50	39.31	0.54	0.67	0.71	0.78	2.63	3.35	2.39	2.90	4.90	5.01	3.38	3.72
6	33.58	35.81	37.02	37.07	1.00	0.92	1.24	1.00	4.55	4.14	4.65	4.23	4.53	4.52	3.75	4.23
7	31.78	34.06	35.37	35.77	0.90	0.79	0.72	0.95	5.72	6.15	5.35	5.91	6.38	7.80	7.38	6.22
8	35.12	36.65	38.52	38.25	1.67	1.71	1.36	1.47	3.77	3.00	4.07	3.48	2.26	1.76	2.99	2.37
9	34.21	36.37	37.67	37.49	0.58	0.29	0.38	0.43	3.66	4.24	3.67	3.90	6.31	14.50	9.76	9.07
10	32.42	33.92	35.48	35.72	1.25	1.19	1.63	1.42	3.70	4.00	3.87	4.11	2.97	3.35	2.38	2.63
11	32.53	34.45	36.19	36.24	0.54	0.64	0.48	0.60	1.99	2.06	1.91	1.58	3.70	3.25	3.95	3.74
12	34.28	35.76	37.80	37.72	0.55	0.21	0.35	0.43	1.64	2.16	1.82	1.61	3.00	10.17	5.16	3.74
13	35.70	37.16	39.54	39.14	0.81	1.13	1.16	1.00	1.56	1.74	1.82	1.67	1.91	1.54	1.57	1.67
14	34.59	37.19	38.06	38.42	0.49	0.35	0.57	0.39	3.28	2.77	3.28	3.31	6.70	7.97	5.79	8.49
15	34.10	36.25	37.61	37.84	0.58	0.62	0.63	0.67	4.40	4.30	4.97	4.53	7.57	6.96	7.87	6.75
16	33.53	35.82	37.32	37.40	0.46	0.29	0.31	0.38	3.28	3.36	3.53	3.84	7.11	11.50	11.48	10.10
17	35.11	36.88	38.93	38.61	1.03	0.95	0.93	0.83	3.36	2.86	3.26	2.77	3.26	3.00	3.52	3.34
18	34.41	36.19	38.02	38.00	0.74	0.59	0.69	0.58	2.00	1.63	1.40	1.40	2.69	2.75	2.03	2.42

**Table 5:** Quantification results of WCI impact factors.

No.	S (ha)	LSI	PI	PG	MNNg	MNNi
1	2.78	3.28	0.74	0.25	1.32	0.87
2	4.64	5.71	0.67	0.31	0.54	0.72
3	6.53	1.51	0.50	0.48	2.11	1.06
4	2.57	1.89	0.57	0.43	1.33	1.60
5	2.40	1.32	0.86	0.14	1.16	0.83
6	7.52	1.84	0.66	0.33	1.40	2.05
7	3.28	1.51	0.54	0.43	1.43	2.00
8	1.18	1.81	0.49	0.49	0.76	1.51
9	1.14	2.66	0.68	0.32	0.82	0.82
10	12.18	1.62	0.42	0.58	1.26	1.84
11	3.84	2.20	0.66	0.33	1.23	0.79
12	1.18	3.37	0.87	0.13	0.89	0.86
13	8.66	1.37	0.68	0.19	1.17	1.12
14	1.11	3.48	0.82	0.18	0.92	0.79
15	2.16	2.81	0.72	0.27	0.96	0.99
16	1.25	5.79	0.76	0.24	0.95	1.87
17	2.17	1.98	0.65	0.28	1.11	1.01
18	1.60	2.10	0.75	0.25	1.22	0.42

**Table 6:** Correlation analysis between selected impact factors.

	LSI	PI	PG	MNNg	MNNi
S	−0.334	−0.542 *	0.458	0.412	0.382
LSI		0.396	−0.326	−0.569 *	−0.136
PI			−0.966 **	−0.375	−0.538 *
PG				0.359	0.538 *
MNNg					0.239

Note: ** means *p* < 0.01, * means *p* < 0.05 (*n* = 18).

**Table 7:** Correlation between WCI and impact factors.

	S	LSI	PI	MNNi	MNNg
L	0.509 **	−0.621 **	−0.723 **	0.468 **	0.333 **
ΔT	0.133	−0.017	−0.549 **	0.550 **	0.285 *
G	−0.230	0.620 **	0.150	−0.033	−0.357 **

Note: ** means *p* < 0.01, * means *p* < 0.05 (*n* = 72).

**Table 8:** Regression analysis results for WCI range and impact factors.

	Weight Factors	Quality
Constant	2.050	R^2^ = 0.689 F = 26.59 Significance *p* < 0.01
S	0.014	
LSI	−0.134	
PI	−1.282	
MNNg	−0.220	
MNNi	0.109	

**Table 9:** Regression analysis results for WCI amplitude and impact factors.

	Weight Factors	Quality
Constant	4.691	R^2^ = 0.409 F = 14.289 Significance *p* < 0.01
PI	−3.663	
MNNg	0.247	
MNNi	0.856	

**Table 10:** Regression analysis results of WCI gradient and impact factors.

	Weight Factors	Quality
Constant	0.936	R^2^ = 0.362 F = 17.879 Significance *p* < 0.01
LSI	1.842	
MNNg	−0.027	
